# *In vivo* tissue bioimpedance dataset

**DOI:** 10.1038/s41597-026-07652-y

**Published:** 2026-07-17

**Authors:** Chiara Falagario, Elena De Momi, Leonardo S. Mattos

**Affiliations:** 1 Biomedical Robotics Lab, Department of Advanced Robotics, Istituto Italiano di Tecnologia, Genoa, Italy; 2 Department of Electronics, Information and Bioengineering, Politecnico di Milano, Milan, Italy

## Abstract

This dataset includes *in vivo* electrical bioimpedance data collected from different biological tissues of two male Yorkshire pigs. The tissues probed include muscle, kidney, liver, bladder, intestine, skin, subcutaneous tissue, stomach, tendon, spleen, peritoneum, and blood from different vessels. Measurements were performed using a custom-made electrical impedance analyzer with two different types of sensing electrodes (monopolar and bipolar) and two sensing modalities (single fixed frequency and frequency scan). This data can be used to support a wide range of research and development efforts towards medical technologies that use electrical bioimpedance sensing as a key source of information to support clinical decisions, for example during diagnosis, treatment administration, or surgery. Specifically, this dataset may be used to support the development of technologies for autonomous identification of different tissues on contact, as demonstrated by the illustrative results presented in this paper. This capability may support future research towards assistive systems for enhancing procedural precision, safety and effectiveness, and may contribute to the development of autonomous robotic functionalities.

## Background & Summary

The integration of Electrical Bioimpedance (EBI) sensors into clinical practice is becoming a highly relevant research topic. EBI sensors have been researched and developed in various studies to characterize tissue properties in real-time^1^ and use that information to detect critical moments during clinical tasks, such as venipuncture during intravenous catheterizations^2^, reaching of the cortical bone to avoid breach during pedicle screw drilling^3^, or proximity of a retinal vessel during vitreo-retinal microsurgery^4^. EBI sensors have also been used in the context of diagnosis, for instance to characterize skin neoformations^5^ and to discriminate cancerous from healthy tissue in the upper aerodigestive track^6^. Furthermore, the use of EBI sensors has been investigated to enhance awareness during minimally invasive robotic surgery, demonstrating potential to support the automatic identification of organs^7^, enhanced visualization of abnormal tissue regions^8^, and also to estimate the interaction force between a surgical instrument and tissue^9^. Despite the growing interest in EBI-based technologies, most of the datasets presented in previous studies are limited to a small number of tissue types and report only task-specific experimental data collected to validate individual hypotheses or sensing systems. To the best of our knowledge, no public dataset currently exists that provides a comprehensive collection of *in vivo* EBI measurements acquired across multiple tissue types under consistent experimental conditions. A notable exception is represented by the seminal work of Gabriel *et al*.^10–12^, which provides a thorough review of the electrical properties of biological tissues over a broad frequency spectrum. However, the focus of this series of studies is primarily the theoretical characterization and modeling of tissue electrical behavior, rather than the creation of a standardized and accessible dataset intended for benchmarking purposes.

In this context, our work contributes to advancing knowledge regarding the electrical bioimpedance of tissues by introducing a comprehensive *in vivo* EBI dataset acquired from different biological tissues, which may support future research on EBI-based technologies intended for clinical applications.

The data was collected during surgical procedures on two healthy porcine models, which were performed at the Mini Invasive Advanced Surgery Academy (Milan, Italy) with approval from the Italian Ministry of Health n°654/2023-PR (*Risp. a prot. AC827.8*). Measurements were performed on 14 different types of tissue, including the following: muscle, skin, subcutaneous tissue, tendon, bladder, intestine, peritoneum, liver, colon, kidney, spleen, stomach, and blood from peripheral veins and deep vessels. The whole set of measurements was collected using SmartProbe^1^ with various acquisition modalities, two types of sensing electrodes (monopolar and bipolar) and two sensing modalities (single fixed frequency and frequency scan). Therefore, the dataset provides a broad representation of the bioimpedance of *in vivo* tissues, allowing comparisons between sensing techniques and the validation of the use of EBI for *in vivo* tissue discrimination.

Data processing and classification algorithms were implemented and assessed in this work. Representative experiments suggest that the dataset could support the preliminary development and evaluation of tissue identification tasks. As follows, illustrative example classifiers trained on diverse data combinations successfully distinguished between multiple tissue types and identified the transition point from air to blood during needle insertion into vascular structures.

The proposed dataset constitutes a resource for both the study of *in vivo* tissue electrical characteristics and the development of advanced intraoperative sensing technologies, offering a starting point for future works towards systems for enhancing the precision, safety and effectiveness of medical procedures. Nevertheless, some limitations should also be acknowledged. The dataset has a limited sample size and reflects measurements obtained using a specific custom sensing platform within a restricted frequency range. Future extensions may include additional animal subjects, pathological tissues and broader acquisition conditions.

## Methods

### Ethics statement

The study was approved by the internal ethics board of the Fondazione Mini Invasive Advanced Surgery Academy (MIAS; Milan, Italy), which is a surgical training center authorized and fully equipped for experimentation on vertebrate animals. The study protocol (number AC827.8) was submitted to the Italian Ministry of Health directly by the Fondazione MIAS, obtaining approval with protocol number 654/2023-PR. The study protocol was fully aligned with relevant regulations and accepted guidelines for vertebrate animal studies, such as the EU Directive 2010/63/EU.

### Dataset acquisition

#### Animal models and surgical environment

Two male Yorkshire pigs weighing between 20 to 30 kg, without genetic modification and in good health status, were used for the EBI data collection. This procedure was a secondary objective of a study involving the assessment of a novel navigation system for minimally invasive surgeries^13^. The animals were housed at the MIAS animal facility and allowed a 12-hour acclimatization period before commencing the study protocol. The experiment took place in the MIAS operating room, where the animals received general anesthesia administered by a trained veterinarian.

The animal was placed in a supine Trendelenburg position. Premedication consisted of an intramuscular injection of tiletamine at 4 mg/kg and zolazepam at 4 mg/kg (Zoletil), combined with dexmedetomidine at 10 mcg/kg (Dexdomitor). Intravenous access was obtained in both ear veins using 20-gauge catheters. Following intravenous administration of propofol at 4 mg/kg (Propovet), the animal was intubated and mechanically ventilated with a tidal volume of 10–13 mL/kg. General anesthesia was then maintained throughout the procedure with 2–3% sevoflurane (Sevflo). Analgesia was ensured through continuous intravenous infusion of fentanyl at 30 mcg/kg/h (Fentadon). At the conclusion of experimental procedures, the animal was euthanized according to approved veterinary protocols by intravenous administration of pentobarbital sodium (150 mg/kg). This method ensures a painless procedure, as it rapidly depresses the central nervous system, resulting in loss of consciousness followed by cessation of respiratory and cardiac functions. All anesthesia and euthanasia procedures were performed in compliance with current veterinary best practices. Standard minimally invasive surgery instrumentation was used to perform the navigation system trials. Upon conclusion of those trials, the acquisition of EBI measurements *in vivo* started.

#### SmartProbe acquisition system

**Fig. 1:**
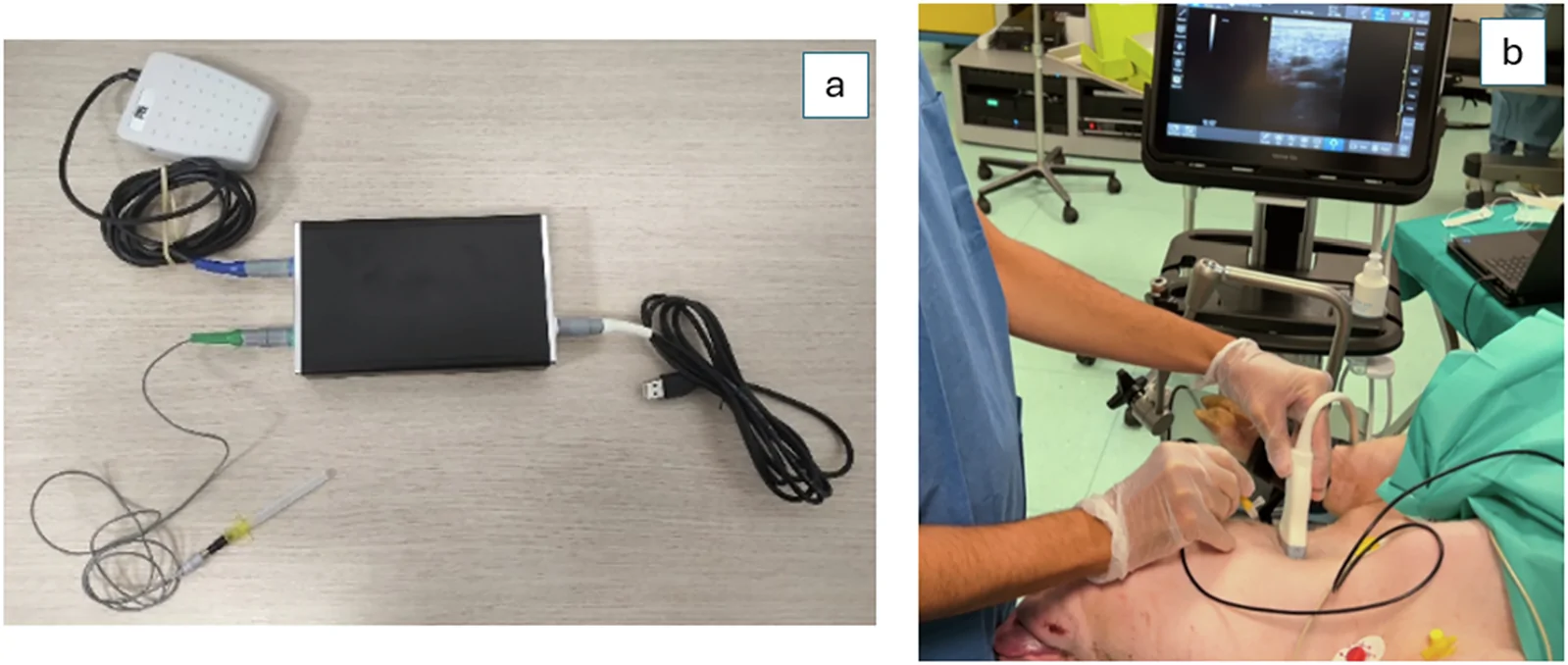
Data acquisition setup. (**a**) SmartProbe system; (**b**) Data acquisition process on porcine tissues.

EBI measurements were performed by an expert surgeon using SmartProbe^1^, a custom system operating in the frequency range from 10 kHz to 100 kHz. The device is depicted in Fig. 1a, alongside a picture of the data acquisition process.

SmartProbe is a needle-based bioimpedance sensing device designed for the characterization of the passive electrical properties of body tissues. The system integrates a disposable concentric needle electrode (CNE) with an Arduino DUE board (Arduino Inc., Italy) and a high precision impedance converter based on the AD5933 (Analog Devices, Norwood, MA, USA) to perform multi-frequency impedance measurements in real time. As reported in previous validation studies, SmartProbe was optimized to minimize measurement variability associated with CNEs manufacturing tolerances through a calibration procedure involving saline solutions. Benchmarking against laboratory-grade impedance spectroscopy instrumentation was partially addressed during the original SmartProbe development and validation phases through calibration procedures and controlled phantom experiments. The reported validation experiments including comparison against calibrated impedance references and phantom measurements demonstrated stable impedance measurements with good repeatability and sensitivity to tissue-dependent dielectric variations, yielding a relative error smaller than 6%. Residual uncertainties were expected to arise from electrode positioning variability, tissue heterogeneity, electrode polarization effects, and environmental noise inherent to intraoperative measurements.

The 10-100 kHz operating range was primarily chosen as application driven. In fact, it provides sufficient sensitivity to biologically relevant impedance contrasts while maintaining adequate signal-to-noise ratio, reduced parasitic effects, and stable electrode-tissue interface behavior during measurements. In this interval, the measured impedance shows almost no dependence from frequency and is predominantly influenced by extracellular and membrane-related electrical properties^14^, which are known to differ between tissues due to variations in cellularity, water content, vascularization, and tissue architecture. Within this bandwidth, the system can reliably estimate bulk resistive and dispersive tissue characteristics relevant for tissue differentiation. However, the selected range does not allow full characterization of higher-frequency dielectric phenomena, such as intracellular polarization mechanisms and complete β-dispersion characterization, which typically require measurements extending into the MHz range. Therefore, the dataset is to be considered primarily suitable for relative tissue discrimination.

#### *In vivo* tissue acquisition procedure

The data was collected using monopolar and bipolar electrode configurations. For each tissue type, measurements were performed by applying the specific frequency sweep of the device in use. Additionally, leveraging the SmartProbe’s functionalities, acquisitions at a fixed excitation frequency were also conducted. Tissue data acquisition was carried out by integrating the acquisition devices with a probe equipped with a disposable sterile needle electrode, selected among the variants shown in Table 1.

**Table 1:** Specifications of the needle electrodes used for signals acquisition.

Assigned name	Producer	Code	Length (mm)	Diameter (mm)	Electrode configuration
Yellow	FIAB	F8990/45	45	0.45	Bipolar
White	FIAB	F8990/65	65	0.45	Bipolar
Monopolar	FIAB	F8993/50	50	0.5	Monopolar

All measurements performed using SmartProbe used *FIAB* needles. For measurements involving the monopolar configuration, a *LUMED ET1119 skin electrode* (GMND-CODE: 35035) was used as the reference electrode, placed on the pig’s chest.

Each acquisition followed a standardized procedure consisting of the following steps:

- Wiping the needle probe using a piece of gauze soaked in 90% denatured ethyl alcohol;
- Calibrating the probe by performing an acquisition in the air, without contact with any objects;
- Measuring the tissue impedance by placing the needle in contact with the target tissue and recording the signal.

During the measurements, the SmartProbe needle electrode was inserted directly into the target tissue. To minimize measurement variability and improve placement consistency, SmartProbe insertion was performed under direct visual guidance with the support of an ultrasound probe (Fig. 1b), allowing more accurate targeting of the tissue region of interest. After insertion, impedance spectra were acquired while maintaining stable probe positioning to minimize motion artifacts and electrode-tissue interface variability. The concentric needle configuration enabled localized impedance sensing within a confined tissue volume surrounding the needle tip, allowing direct interrogation of the tissue of interest during the intraoperative procedure.

All measurements were automatically saved in text files (.txt), which were named by the surgeon with the date and time of the measurement and included metadata describing the tissue type, the acquisition instrument and the needle used. Subsequently, the raw impedance data files were prepared and organized: file names were standardized to include the English name of the tissue probed, the acquisition mode and configuration employed.

### Data preparation

The original dataset contained a total of 70 data files and was processed and analyzed using MATLAB R2025b. Each file was loaded in the workspace and underwent a preliminary phase to validate structural integrity: this included verifying schema consistency, the presence of all required metadata fields and the correctness of acquisition parameters. Range checks were performed to ensure all frequency values were within the expected limits for the specific acquisition mode. Files with missing values or structural anomalies were excluded, resulting in a final dataset of 56 valid files (Table 2).

**Table 2:** Overview of the composition of the final dataset described in this work.

	skin	subcutaneous tissue	tendon	muscle	bladder	intestine	peritoneum	liver	kidney	spleen	stomach	vein	artery
**Pig 1**	2	4	3	7	2	2	1	2	1	1	1	12	8
**Pig 2**	0	0	0	0	0	0	0	1	0	0	0	6	3
**Total: 56**	**2**	**4**	**3**	**7**	**2**	**2**	**1**	**3**	**1**	**1**	**1**	**18**	**11**

#### SmartProbe data conversion and correction

The original data recorded using SmartProbe corresponded to the raw impedance data measured by its integrated AD5933 chip. This raw data should undergo a conversion procedure to represent actual impedance values, as described in detail in the AD5933 datasheet. Here, we performed this data conversion procedure with Gain Factor and System Phase parameters obtained by calibrating the system with a known 20 kΩ resistor.

In addition, as some parts of the dataset contain data acquired using different concentric electrode needles (CENs) mounted on the SmartProbe, a correction process was implemented to enable a proper comparison between these data. For this, *Yellow* and *White* electrodes (as described in Table 1) were used to acquire calibration signals using a 0.9% sodium chloride solution, also known as physiological solution. The resulting measurements were processed to estimate, for each frequency, a complex transfer factor G(f) describing the relationship between the impedances measured with the two needles, according to ${Z}_{{yellow}}\approx G(f){Z}_{{white}}$. The algorithm reads the two calibration files, removes outliers and invalid samples, and converts the raw data into magnitude, phase, and real/imaginary impedance components, correcting for system gain and phase within the 10–100 kHz frequency range. For each common frequency, the complex transfer factor G(f) is then estimated through a least-squares approach, as shown in (1).

1 $$G\left(f\right)=\frac{{Z}_{{yellow}}^{H}{Z}_{{white}}}{{Z}_{{white}}^{H}{Z}_{{white}}}$$

The resulting structure includes: the reference electrode *Yellow*, the frequency vector, the complex transfer function G(f) and the number of valid pairs per frequency, along with metadata on the source file. In the subsequent data preparation step this calibration structure is used to harmonize all measurements to a common reference needle. Concretely, any impedance dataset acquired with the *White* electrode is transformed into the equivalent *Yellow* electrode measurement by applying the estimated transfer function. The calibration transfer function G(f) is stored together with its frequency grid and metadata and is then applied to each measurement ${Z}_{\mathrm{white}}(f)$ to obtain the corrected value.

2 $${\hat{Z}}_{\mathrm{yellow}}(f)=G\left(f\right){Z}_{\mathrm{white}}\left(f\right)$$

The correction in (2) ensures that all subsequent analyses are performed on impedance data expressed with respect to the same needle characteristics.

The corrected real and imaginary parts of the impedance data are then used to recompute its magnitude and phase. In addition, the acquisition time is normalized to $[\mathrm{0,1}]$. If no valid transfer map is found, the data is left unchanged, and an informative warning is issued. This procedure standardizes all measurements to the same needle reference, enabling consistent comparison across different electrodes. Users can reproduce this process by applying the parameters saved in the *Tissue Analysis Scripts* folder.

The final dataset consists of the calibrated and corrected signals; nevertheless, the original raw signals can also be accessed through the container “*OriginalData*” inside the dataset’s *Supplementary* folder. The processed data is represented by matrices that contain frequency, corrected magnitude and phase, as well as the corresponding real and imaginary parts of the impedance, preserving any additional experimental metadata from the original dataset. The processing step just described ensured that the data reflects accurate electrical impedance measurements, ready for subsequent analysis or modeling.

## Data Records

The *in vivo* porcine tissue bioimpedance dataset^15^ is available at Zenodo under (https://doi.org/10.5281/zenodo.18769576). The dataset is structured into a hierarchical folder order based on animal subject, measurement configuration, acquisition type, and histological group. The dataset contains text files storing impedance measurements. Each file includes a metadata header describing the acquisition setup and device configuration, followed by columns containing the measurement mode and the recorded EBI values.

**Fig. 2:**
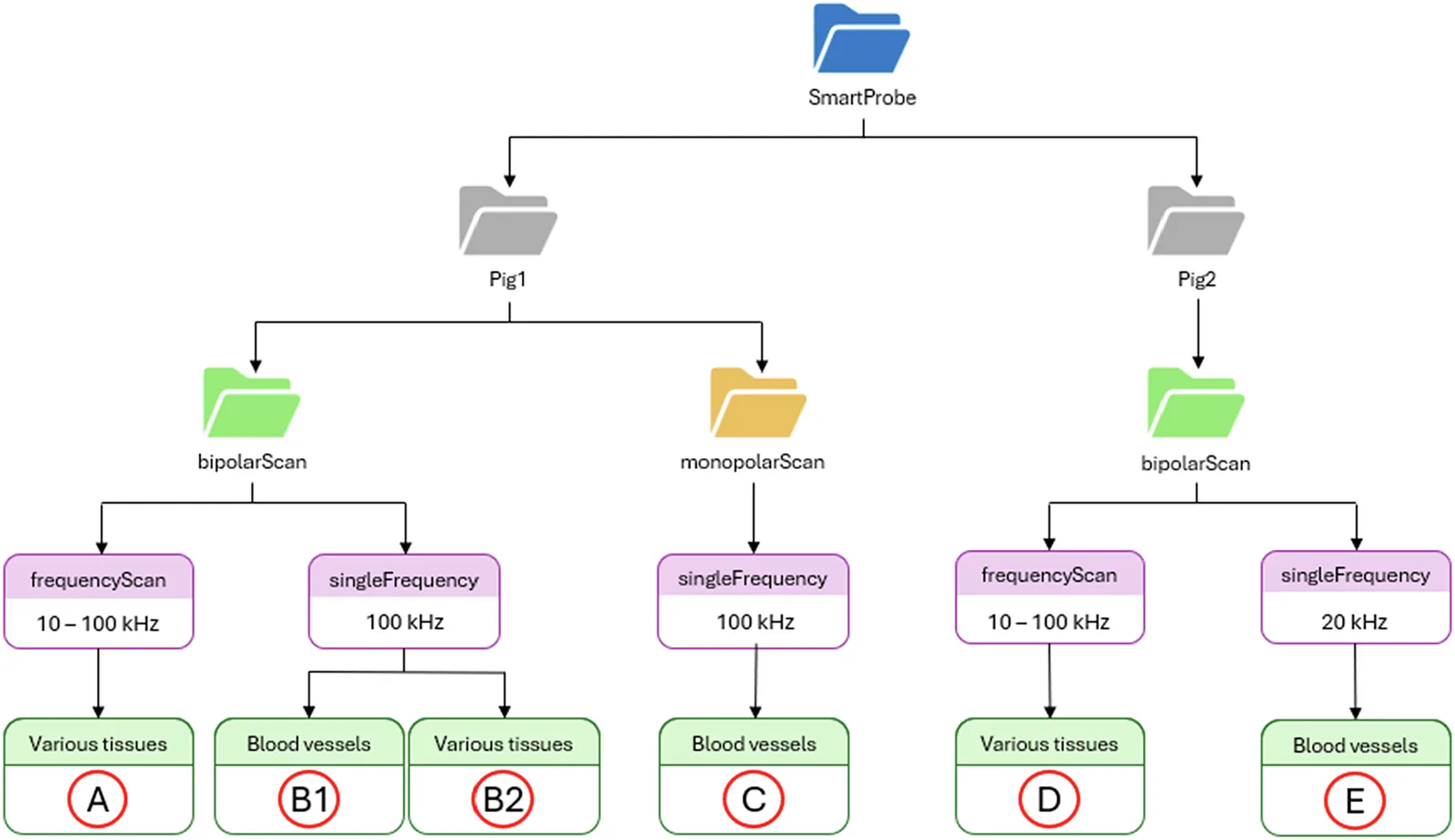
Directory structure of the *in vivo* EBI dataset. The dataset is hierarchically organized according to animal subject (grey folders), measurement configuration (green folders for bipolar acquisitions and yellow folders for monopolar acquisitions), acquisition type (purple boxes) and histological category (green boxes). Each dataset branch (subset) is identified by an alphanumeric code (A-E) to facilitate dataset navigation and cross-referencing throughout the manuscript.

The overall dataset structure is summarized in Fig. 2. To facilitate dataset navigation and cross-referencing throughout the manuscript, each populated subset of the dataset is defined by an alphanumeric code (A-E). In this work, the term “subset” refers to a specific combination of the categories listed above.

The root directory *SmartProbe* contains measurements acquired from two different animal subjects: *Pig1*, comprising 46 measurements, and *Pig2*, comprising 10 measurements. The dataset structure is organized as follows:

- ***Pig1:*** contains all signals acquired on the first animal subject. This subset presents: measurements acquired in monopolar and bipolar modalities, measurements acquired using a frequency scan and a single excitation frequency, signals from blood vessels and various tissues.
- ***Pig2:*** contains all signals acquired on the second animal subject. This subset presents: measurement acquired in bipolar modality, measurements acquired using a frequency scan and a single excitation frequency, signals from blood vessels and various tissues.

Each subject folder includes data from bipolar and/or monopolar configurations, organized into:

- ***bipolarScan***: contains measurements acquired at different frequencies (*FrequencyScan* data) and time-domain recordings acquired at a fixed excitation frequency (*SingleFrequency* data).
- ***monopolarScan***: available only for Pig1. This branch contains only *SingleFrequency* data acquired at 100 kHz from blood vessels.

Table 3 provides a detailed summary of the content of subsets illustrated in Fig. 2.

**Table 3:** Definition of the content of the subsets defined inside the proposed *in vivo* tissue bioimpedance dataset.

Subset code	Animal subject	Measurement configuration	Acquisition type	Histological category
**A**	*Pig1*	bipolarScan	frequencyScan	Various tissues
**B1**	*Pig1*	bipolarScan	singleFrequency	Blood vessels
**B2**	*Pig1*	bipolarScan	singleFrequency	Various tissues
**C**	*Pig1*	monopolarScan	singleFrequency	Blood vessels
**D**	*Pig2*	bipolarScan	frequencyScan	Various tissues
**E**	*Pig2*	bipolarScan	singleFrequency	Blood vessels

Monopolar acquisitions are not largely available in the dataset because they were considered an additional acquisition modality. Indeed, past literature studies^16^ showed slightly worse results in the determination of tissue types with monopolar signals. These are generally more sensitive to superficial tissue properties and provide less volumetric information compared to bipolar measurements, which better capture the electrical characteristics of deeper tissue volumes. Although Pig1 and Pig2 do not contain identical acquisition branches, the two subject datasets should be considered complementary rather than strictly equivalent. Pig1 was designed to include a broader range of acquisition configurations, including both bipolar and monopolar measurements, in order to explore different acquisition modalities and tissue conditions. Pig2, instead, provides an additional subset acquired in the bipolar configuration. Therefore, the two animals’ subsets are not intended to provide a one-to-one correspondence across all branches, but rather to collectively extend the variability and biological coverage of the dataset.

**Fig. 3:**
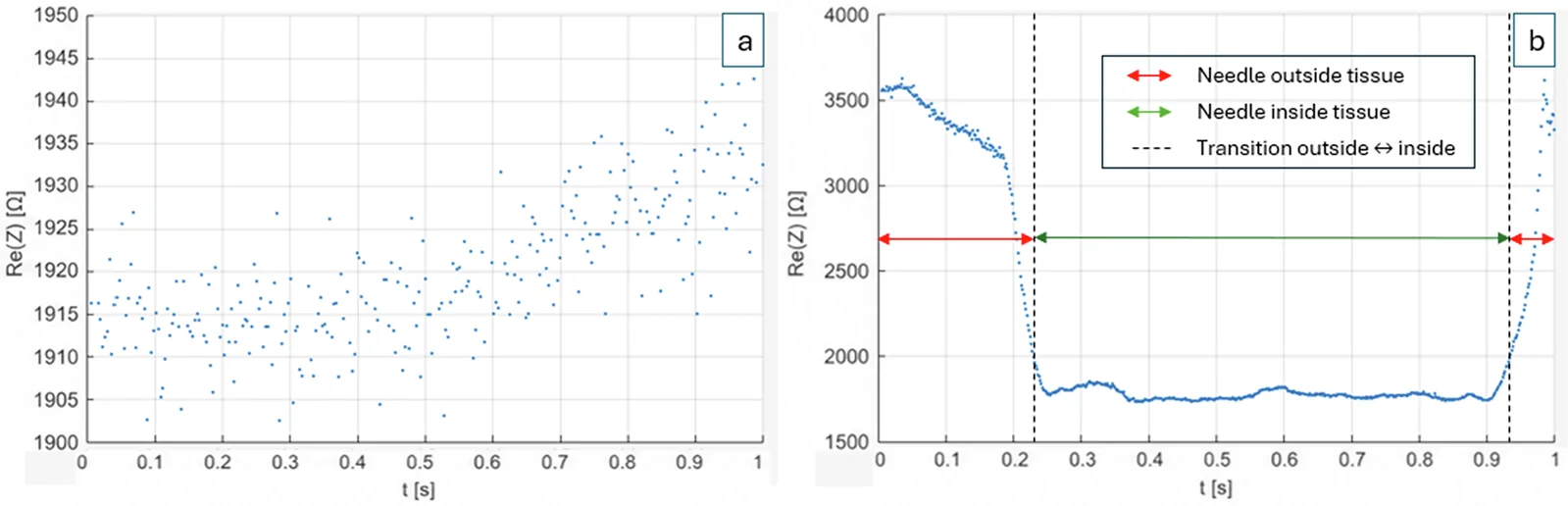
Representative examples of time-domain characteristics of a “vein” impedance signals from subset “C”. (**a**) Real part of the impedance signal acquired when the needle probe is inserted into the tissue and maintained in a stable position; (**b**) Real part of the impedance signal acquired during needle movement inside and outside of the tissue. Red double arrows indicate regions where the needle was outside the blood vessel, whereas green double arrow indicates regions where the needle was inserted in the blood vessel. Vertical dashed lines indicate the instants of transition between outside and inside tissue conditions.

During the acquisitions, the signals were recorded keeping the needle electrode still and inserted inside of the tissue under test for the whole measurement; this type of recording offers an overview of the signals’ spectral properties. On the other hand, data from blood vessels acquired using fixed-frequency modality were collected during the insertion of the needle probe into the tissue. During these acquisitions, the needle trajectory started and/or concluded outside the animal’s body. Thus, the recorded signals show a particular envelope in the time domain, directly associated with the transition of the probe between inside and outside the tissue. Representative examples of these characteristics are shown in Fig. 3. The impedance values were obtained after a calibration and correction procedure designed to minimize the influence of the measurement chain, electrode parasitic, contact impedance, and acquisition-related offsets on the recorded impedance values. Nevertheless, differences between acquisition modalities (e.g., monopolar versus bipolar configurations) are expected due to the different electric field distributions and sensing geometries associated with each acquisition setup. In particular, Fig. 3b reports the real part of the impedance signal acquired from a “vein” tissue included in subset “C”. The higher impedance values correspond to the needle outside the vessel, whereas the lower and more stable impedance region corresponds to the needle inserted inside the vein. In the specific subsets of data, the recordings were performed using different electrode needles, which were replaced between one acquisition and the following one. This approach made it possible to assess how the various needle properties, such as diameter or length, affected the signals obtained. To allow for the comparison of data coming from different electrodes, the FIAB F8990/45 was used as a reference. More insights into these processes are reported in the “SmartProbe Data Conversion and Correction” section above.

Each data file is saved in .txt format and is composed of a header in comment format (preceded by %), which specifies the acquisition device and configuration, and by columns reporting the measurement mode and recorded EBI values. Table 4 lists all the information contained in the header part of the data files.

**Table 4:** Details on the metadata contained in the header of data files acquired with SmartProbe.

Device name and version
**Manufacturer data**
**Data structure description:** **a. Device State:** The state or mode of the device during the measurement. Value “1” indicates frequency sweep acquisition. Value “2” indicates single fixed frequency acquisition. **b. Scan Frequency (kHz):** The excitation frequency used for that measurement, expressed in kHz. **c. Raw EBI (real part):** Raw value of real component of the measured electrical bioimpedance. **d. Raw EBI (imaginary part):** Raw value of imaginary component of the measured electrical bioimpedance. **Time Stamp:** Time since the start of acquisition (internal microcontroller time), expressed in milliseconds.

The data is structured according to the acquisition mode indicated by the Device State field. When the Device State is set to “1” (frequency scan mode), the file contains data blocks corresponding to distinct excitation frequencies, ranging from 10 kHz to 100 kHz in 10 kHz increments. Each block includes multiple consecutive measurements at a specific frequency, enabling noise reduction, e.g. by averaging. Conversely, when the Device State is set to “2” (fixed frequency mode), all data points refer to a determined frequency value, and acquisition is performed continuously over time. In both cases, the measurements represent complex impedance values over time, allowing detailed temporal and spectral analysis for each tissue sample.

### Supplementary scripts

The dataset is structured to support a range of research topics and data analysis methods, including multivariate frequency-domain signal analysis, repeatability evaluation, classification model development and benchmarking of impedance-based tissue sensing systems. A set of MATLAB scripts is provided in the “*Tissue Analysis Scripts*” folder to assist users in loading, processing and visualizing the data. These scripts are intended as a reference implementation and can be adapted for custom analyses. The contents of all scripts and functions, categorized by dataset and specific tasks, are described below.

#### “_DATA_PROCESSING”

This folder contains scripts for conversion and correction of data before analysis:

- ***Data_Freq_Processing.m*:** Conversion and saving to file of data collected with the *SmartProbe* in the frequency domain.
- ***Data_Time_Processing.m*:** Conversion and saving to file of data collected with the *SmartProbe* in the time domain.
- ***Data_Time_Processing_multineedle.m*:** Conversion, electrode-based correction and saving to file of data collected with the *SmartProbe* in the time domain using multiple CENs.
- ***SmartProbe_EBIData_Preparation:*** Preprocessing of raw data collected in the original dataset.
- ***yellow_saline_0.9_NeedleTransfer_Yref:*** File containing the complex transfer factor G(f) mapping the relationship between the impedances measured with two needles.

#### “_ANALYSIS_&_TASKS”

This folder contains scripts for dataset analysis and implementation of tasks:

- ***detect_vesselPuncture.m:*** Implement the threshold-based blood detection algorithm.
- ***SmartProbe_EBIData_Analysis.m*:** Perform the complete analysis of data acquired with the SmartProbe.
- ***smote_balanced.m*:** Implement the SMOTE-based algorithm for balancing class distributions in classification tasks.
- ***SP_Classification.m:*** Perform smoothing and class balancing before training classifiers.
- ***SP_DataAnalysis.m****:* Perform data validation tasks, including summarizing plots, metrics computation and statistical analysis.

#### “_UTILITIES”

This folder contains supporting functions to the main scripts for data processing and analysis:

- ***computation_needleTransfers.m:*** Compute complex transfer factor $G\left(f\right)$ using calibration signals acquired in isotonic saline solution (NaCl 0.9%).
- ***wrapper_DataCleaner.m:*** Visualize signals’ envelopes (one file at a time) and choose regions of the signals to discard or keep.
- ***evaluate_detect_vesselPuncture****.***m:** Validate and evaluate the threshold-based blood detection algorithm.

Users are encouraged to review the metadata headers in the raw .txt files to understand acquisition configurations and measurement structure. All scripts are documented with inline comments to facilitate reuse and adaptation.

## Technical Validation

### Data quality assessment

An assessment of the dataset quality was conducted to evaluate the consistency, repeatability, and physiological plausibility of the acquired bioimpedance measurements across different porcine tissues. The workflow integrated multiple complementary analyses.

#### a) Scatter plots and group visualizations

Scatter plots and grouped visualizations of resistance, reactance and magnitude distributions were generated to verify that the recorded data followed physiologically plausible trends and that the dataset was free from gross anomalies or misclassifications. These analyses provided an initial qualitative assessment of the overall consistency and distribution patterns of the impedance measurements across tissues and frequencies. Visual inspection of the scatter plots did not reveal major abnormal behaviors. The measurements exhibited a decreasing frequency-dependent behavior, coherently with the dielectric properties of biological tissues described in literature^14^ and with the expected reduction of membrane polarization effects at higher frequencies. In particular, tissues characterized by higher water and ionic content (e.g., arterial blood) showed lower resistance values and faster impedance decay across frequency, whereas denser or less hydrated tissues (e.g., kidney and muscle) exhibited higher impedance levels, and more pronounced reactive behavior. Tissues like liver and intestine displayed broader variability patterns, likely reflecting their intrinsic structural heterogeneity, vascularization, and hydration variability. Group visualizations confirmed clear separation among several tissues, indicating that the acquisition system captured tissue-specific electrical signatures with good stability. Scatter plots allow detection of outliers, measurement artifacts or unexpected clusters. Scatter plot inspection did not reveal major abnormal clusters or systematic distortions, while group visualizations confirmed clear separation among tissue categories, supporting both the reliability of the acquisition process and the physiological consistency of the recorded dataset.

#### b) Quantitative impedance parameters

Quantitative impedance parameters were extracted and summarized to further evaluate the physiological relevance of data and verify whether different acquisitions behaved coherently and follow expected trends. The computed quantitative indicators are the following:

- *Reactance to resistance ratio* (indicated as *X/R ratio*), a dimensionless indicator of the relative contribution of capacitive and resistive effects in biological tissues at different frequency points, provides insight into cell membrane integrity, tissue composition and dielectric behavior. This metric also enables the identification of unusual tissue responses, measurement distortions or frequency-dependent inconsistencies. The X/R ratio showed frequency-dependent trends coherent with the capacitive nature of biological membranes. Arterial tissue, for example, exhibited highly negative X/R values at low frequencies that progressively approached zero at higher frequencies, reflecting the expected reduction of membrane polarization effects. Similar monotonic trends were observed across all tissue categories, indicating stable dielectric behavior and absence of major frequency-dependent distortions.
- *Normalized impedance magnitudes* across frequencies allow comparison between different acquisitions, exposing deviations that may indicate errors during the acquisition and/or pre-processing steps. Normalized impedance magnitude analysis across frequencies revealed homogeneous spectral profiles within the same tissue group and distinguishable responses between tissues.
- *Non-parametric statistical comparisons* based on Kruskal–Wallis post-hoc analysis identified significant differences between several tissue pairs, confirming the discriminative capability of the acquired impedance data. Significant separations were particularly evident between tissues with great histological differences (e.g., skin and kidney, p < 0.001), while tissues with similar structural composition showed partially overlapping distributions (e.g., muscle and intestine).

#### c) Interquartile ranges

Interquartile ranges (IQR) analysis was performed to ensure statistical reliability and identify potential outliers or unstable acquisition regions. The IQR provides a robust estimate of data dispersion and is particularly useful for detecting heterogeneous responses without excessive sensitivity to isolated extreme values. Most tissues exhibited moderate dispersion levels within 20% of the median impedance magnitude, indicating stable and repeatable measurements across acquisitions. Larger IQRs were found in tissues (e.g., liver and intestine) having intrinsic biological heterogeneity and higher sensitivity to hydration variability. Importantly, no abrupt or irregular variability increases were observed across the frequency range, suggesting that the acquired dataset was globally stable and free from severe noise contamination or systematic acquisition instabilities.

#### d) Confidence intervals

Confidence interval analysis further supported the repeatability of the measurements. Narrow 95% confidence intervals were observed for most tissues and frequency ranges, particularly for resistance and impedance magnitude measurements, indicating good repeatability across repeated acquisitions. For example, arterial tissue exhibited relatively low coefficients of variation for impedance magnitude (typically below 10% across most frequencies), whereas tissues such as vein and liver showed wider dispersion due to their more heterogeneous morphology and physiological variability.

The combined interpretation of various statistical measures, analyses and controls supports the idea that the acquired bioimpedance dataset was physiologically plausible, statistically reliable, and sufficiently discriminative to distinguish among different porcine tissue types across the investigated spectral range.

### Illustrative validation experiments

Electrical properties of biological materials vary according to the fluid content of the material. For example, blood and brain show high electrical conductivity, lungs, skin, fat, and bone show poor conductivity, whereas liver, spleen, and muscle are intermediate conductors^10–12^. Given the presence of these physiological differences, this section presents illustrative examples of validation experiments intended to demonstrate the dataset usability and provide baseline analyses. Specifically, two sets of experiments based on EBI sensing are described: (1) Automatic Tissue Identification experiments and (2) Venipuncture Detection experiments. Given the availability of data acquired using different devices and sensing modalities, a total of six experiments is described. These are listed in Table 5, which also provides information on the data subsets used in each of them (those were previously defined in Fig. 2).

**Table 5:** List of experiments performed with data from the *in vivo* tissue bioimpedance dataset.

#	Experiment Set	Experiment Name	Subsets involved
1	Automatic Tissue Identification	*Tissue Identification with SmartProbe Frequency Scan*	A, D
2	Automatic Tissue Identification	*Tissue identification with SmartProbe Single Frequency*	B2
3	Venipuncture Detection	*Blood Vessel Puncture Detection with Bipolar Electrodes*	B1, E
4	Venipuncture Detection	*Blood Vessel Puncture Detection with Monopolar Electrodes*	C
5	Automatic Tissue Identification	*Recognition of Arterial versus Venous Blood with Bipolar Electrodes*	B1, E
6	Automatic Tissue Identification	*Recognition of Arterial versus Venous Blood with Monopolar Electrodes*	C

#### Data smoothing and class balancing

For the experiments described in this paper, we conducted additional data processing steps to reduce noise and create balanced datasets for subsequent analysis. The MATLAB function *smoothdata* was used to filter and smooth the time-series data. To address class imbalance, a custom function based on the Synthetic Minority Over-sampling Technique (SMOTE)^17^ was implemented and applied only to the train set within each iteration. This custom SMOTE method extends the traditional SMOTE approach combining over-sampling and under-sampling, ensuring a fully balanced dataset even when the original class distribution is strongly skewed. The first step consists in the generation of synthetic observations for the minority classes, which is done by interpolating existing samples with their nearest neighbors. Then, the function recomputes the class distribution and sets the target size equal to the size of the smallest class; for each class, it counts the current number of samples, *n_current*. If this number is greater than the target size, the class is considered majority and is randomly undersampled to match. If *n_current* is smaller than the target size, no reduction is applied to that class.

#### Development of data classifiers

All experiments presented in this work rely on the classification of EBI data into different tissue target classes; therefore, each experiment required the development and training of supervised classifiers. Model development was carried out using MATLAB R2025b. The definition of an appropriate feature set was guided by previous analyses (“Data Quality Assessment”), which demonstrated that electrical properties were physiologically meaningful descriptors of tissue-dependent impedance behavior.

For fixed-frequency acquisitions, the feature space included magnitude, phase, resistance, and reactance. Since these measurements provide a compact representation of the electrical response at a single frequency, the resulting classification problem was characterized by a low-dimensional feature space. In this context, the Fine K-Nearest Neighbor (KNN) model was selected. This choice was supported by methodological and biological considerations. From a methodological perspective, KNN models are well-suited to low-dimensional datasets where class separation is mainly governed by local proximity relationships. From a biological perspective, the data quality analysis showed that tissue groups formed distinguishable but partially overlapping impedance distributions, especially for tissues with similar composition. Therefore, an instance-based classifier was appropriate because it can capture local differences between neighboring tissue clusters without imposing a rigid global decision boundary. The fine variant was particularly suitable because it is more sensitive to subtle local differences in the feature space. Preliminary model comparisons performed in the Classification Learner app confirmed that Fine KNN provided higher classification accuracy and lower error than alternative models for the frequency-fixed datasets.

For frequency-scan acquisitions, the frequency value was included together with magnitude, phase, resistance, and reactance in the feature space. This produced a richer representation that incorporated the spectral evolution of each tissue response. The previous analysis showed that electrical measures followed tissue-specific frequency-dependent trends, reflecting differences in hydration, ionic content, membrane polarization, and structural organization. As a result, class separation was not expected to be purely linear. For this reason, a Cubic Support Vector Machine (SVM) model was selected. Nonlinear SVMs are well suited to datasets in which classes are separated by complex boundaries in a multidimensional feature space. The cubic kernel provides sufficient flexibility to model nonlinear relationships between impedance parameters and frequency while avoiding the excessive complexity and risk of overfitting associated with higher-order kernels. This choice was also supported empirically: during model selection, Cubic SVM outperformed linear, quadratic and other nonlinear SVM classifiers, achieving the highest cross-validated accuracy and the most stable class separation.

Model performance was assessed using a stratified 5-fold cross-validation strategy. For each fold, the dataset was divided into training and validation partitions, with four folds used for training and the remaining fold used for validation. This procedure was repeated five times so that each fold was used once as validation data. To address class imbalance, the SMOTE-based algorithm introduced in “Data Smoothing and Class Balancing” was applied only to the training partition of each fold, while the corresponding validation partition remained unchanged. Feature standardization was also performed within each fold using the mean and standard deviation computed from the training data only, and the same transformation was then applied to the validation data.

#### Performance assessment

The performance of the data classifiers was quantified both at fold level and globally. For each fold, it was evaluated using Accuracy, Precision, Recall, Specificity, F1-score, Cohen’s kappa coefficient, and Matthew’s Correlation Coefficient (MCC). Accuracy was computed as the proportion of correctly classified samples over the total number of samples. Precision was calculated as the ratio between true positives (TP) and the sum of true positives and false positives (TP + FP), while Recall was defined as TP divided by the sum of TP and false negatives (TP + FN). The F1-score was calculated as the harmonic mean of Precision and Recall. Cohen’s kappa coefficient was used to assess classifier agreement beyond chance at each fold, according to (3), where ${p}_{o}$ represents the observed agreement and ${p}_{e}$ the expected agreement by chance. MCC was computed as a balanced performance metric accounting for all elements of the confusion matrix and generalized to the multiclass setting. This metric ranges from -1 (complete disagreement) to + 1 (perfect classification), with 0 indicating random prediction performance.

3 $$\kappa =\frac{{p}_{o}-{p}_{e}}{1-{p}_{e}}$$

Confusion matrices from all folds were then aggregated to obtain global performance metrics and class-wise results for each tissue type inside the subset under test. Finally, bootstrap resampling of the cross-validation predictions was used to estimate 95% confidence intervals for the main validation metrics. Confidence intervals were computed using a percentile bootstrap approach based on 1000 resampling iterations performed with replacement on the predicted labels.

Building on the findings in the previous sections, this assessment reinforces that the electrical bioimpedance signatures captured across the examined tissues follow coherent physiological trends and maintain clear inter-tissue distinctions. The confirmed statistical variability, together with the consistent performance of multiple classification approaches across sensing modalities and electrode configurations, indicates that the dataset reliably represents the underlying biophysical differences observed *in vivo*. The results underline the suitability of measurements for supporting further methodological development, benchmarking and validation of tissue-discrimination strategies based on electrical bioimpedance sensing. The results for each experiment are reported in the following sections.

#### Experiment 1: Tissue Identification with SmartProbe Frequency Scan

The goal of this experiment was to investigate if the EBI data acquired using SmartProbe’s frequency scan functionality could be used to identify different types of tissues *in vivo*. Having comparable SmartProbe frequency-scan signals available from the two porcine subjects, subsets “A” and “D” were combined to create an experimental subset including all represented tissue classes. A Cubic SVM classification model was trained and evaluated on this combined dataset. Although subsets “A” and “D” originated from different porcine subjects, a strict train-validation separation between the two subsets was not considered appropriate for this experiment. Some tissue classes present in subset “D” were either missing or only sparsely represented in subset “A” and vice versa, resulting in an unbalanced class distribution and insufficient representation of the full tissue variability during training. To avoid introducing a bias related to incomplete class coverage rather than true inter-subject variability, the classification analysis was therefore conducted on the combined dataset obtained from subsets “A” and “D”. This approach allowed a more comprehensive representation of tissue-specific bioimpedance characteristics and enabled a more reliable evaluation of tissue discrimination performance.

**Fig. 4:**
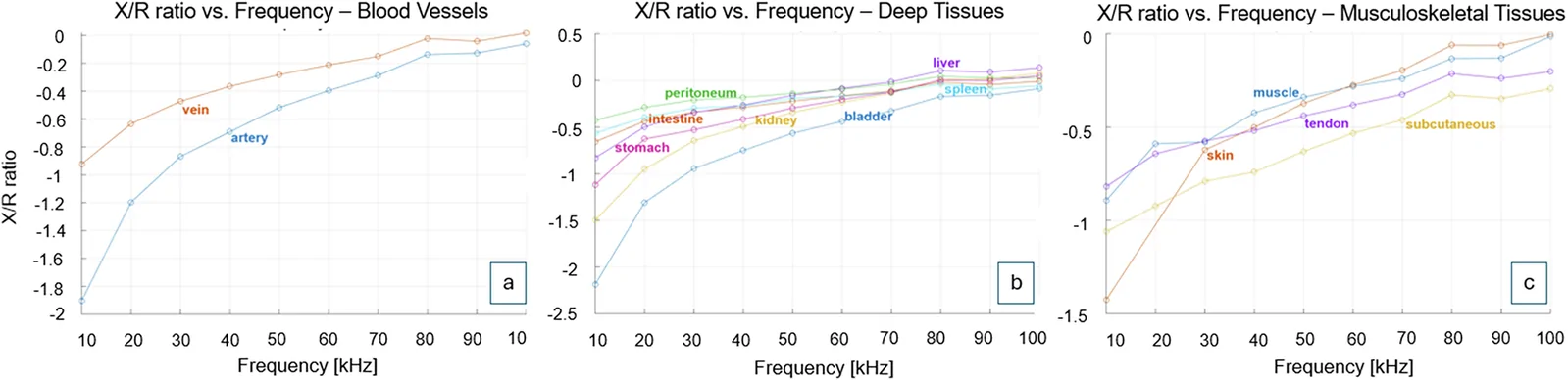
X/R ratio plots of data in the subsets “A” and “D” varying with frequency. Three groups of tissues are defined to facilitate data visualization: (**a**) Blood vessels: artery, vein; (**b**) Deep tissues: bladder, intestine, kidney, liver, peritoneum, spleen, stomach; (**c**) Musculoskeletal tissues: muscle, skin, subcutaneous tissue, tendon.

To facilitate the visualization of the data contained in this experimental set, three groups of tissues were defined: Blood vessels, Deep tissues, Musculoskeletal tissues. Figure 4 highlights distinct frequency-dependent X/R ratio trends at all frequency points for all tissues, according to the group they belong to.

Blood vessels (Fig. 4a) exhibited the lowest absolute X/R values at low frequencies, particularly arterial blood, reflecting the highly conductive nature of blood associated with its elevated ionic and fluid content. The progressive increase of the X/R ratio with frequency suggests a reduced influence of membrane polarization effects as excitation frequency increases. Deep tissues (Fig. 4b) showed intermediate electrical behavior with smoother variations across frequencies. These tissues are characterized by substantial vascularization and relatively high water content, which contribute to moderate conductivity and capacitive behavior. In particular, liver and spleen displayed comparatively higher X/R ratios at higher frequencies, likely reflecting their dense vascular architecture and rich extracellular fluid distribution. Musculoskeletal tissues (Fig. 4c) demonstrated greater variability, consistent with their heterogeneous structural composition. Muscle tissue showed comparatively higher conductivity due to its elevated electrolyte and water content, together with the anisotropic organization of muscle fibers that facilitates current propagation. Conversely, subcutaneous tissue exhibited lower X/R ratios across the explored frequency range, likely due to its high lipid content and reduced water concentration, both associated with poor electrical conductivity. Tendon and skin displayed intermediate behaviors, which may be associated with their collagen-rich fibrous structure and lower vascularization. In the case of skin, the presence of the stratum corneum may additionally contribute to increased impedance and capacitive effects, particularly at lower frequencies.

Overall, the observed trends confirm that bioimpedance measurements are strongly influenced by tissue composition, fluid distribution and dielectric properties, supporting the feasibility of distinguishing tissue types through electrical bioimpedance sensing.

**Fig. 5:**
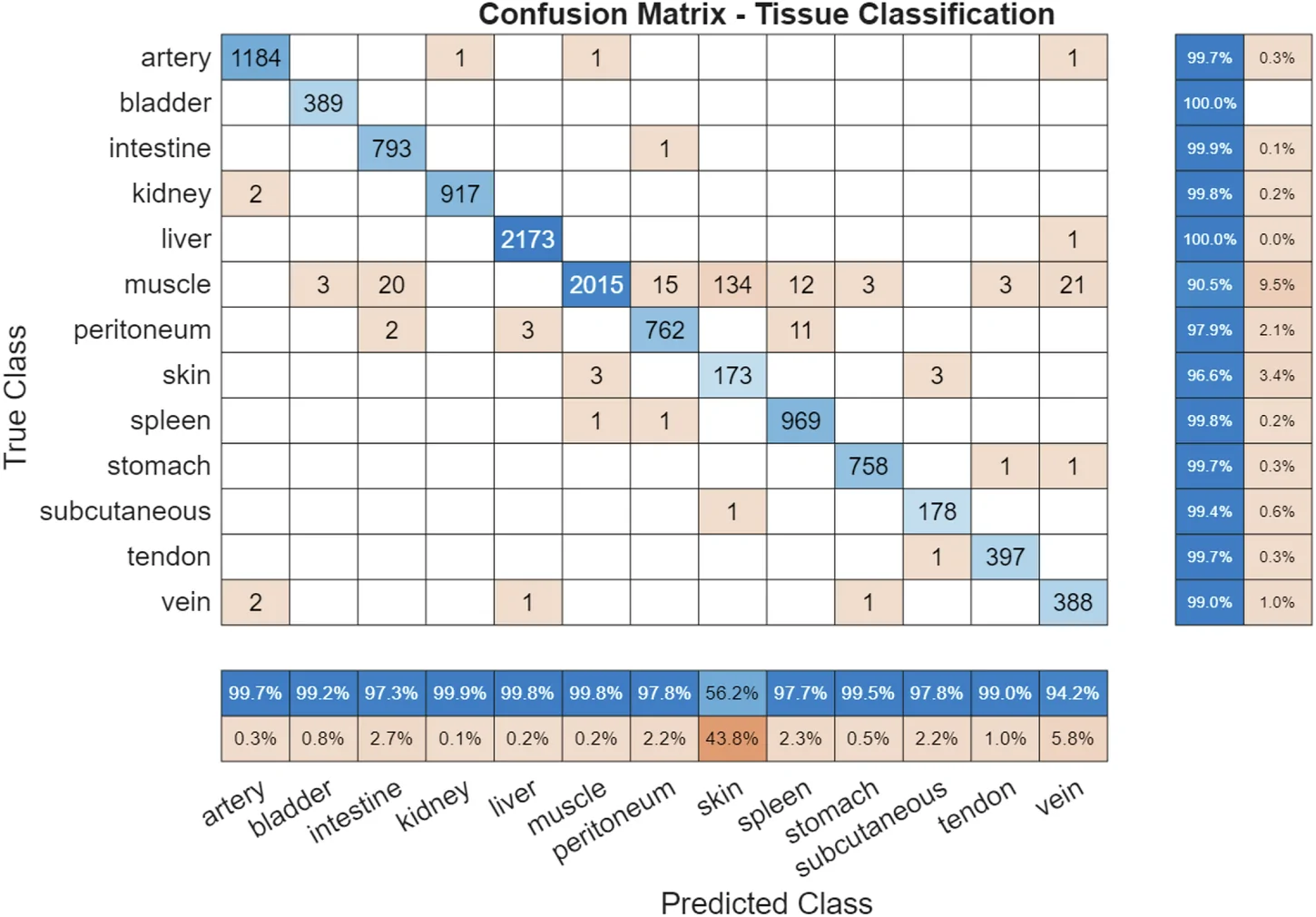
Confusion matrix resulting from tissue identification experiment based on SmartProbe data in the experimental subset.

A Cubic SVM model was trained on data from Pig1 subset “A” and Pig2 subset “D” to correctly identify different tissues. Table 6 and Table 7 show the performance metrics computed for each class and overall, respectively. Figure 5 illustrates the results on tissue classification for this experiment.

**Table 6:** Class-wise performance metrics of the Cubic SVM classifier trained on the combined SmartProbe dataset (subsets “A” and “D”).

Class	Samples	Precision	Recall	Specificity	F1_score
*artery*	1187	0.9992	0.9966	0.9999	0.9979
*bladder*	389	0.9949	1	0.9998	0.9974
*intestine*	794	0.9730	0.9975	0.9979	0.9851
*kidney*	919	0.9978	0.9902	0.9998	0.9940
*liver*	2174	0.9968	0.9954	0.9992	0.9961
*muscle*	2226	0.9946	0.9137	0.9988	0.9525
*peritoneum*	778	0.9757	0.9807	0.9982	0.9782
*skin*	179	0.5897	0.9553	0.9893	0.7292
*spleen*	971	0.9739	0.9979	0.9975	0.9858
*stomach*	760	0.9947	0.9961	0.9996	0.9954
*subcutaneous*	179	0.9727	0.9944	0.9996	0.9834
*tendon*	398	0.9975	0.9975	0.9999	0.9975
*vein*	392	0.9238	0.9898	0.9971	0.9557

**Table 7:** Overall validation performance metrics of the Cubic SVM classifier computed using 5-fold cross-validation predictions on the combined SmartProbe dataset (subsets “A” and “D”). In the right column, 95% confidence intervals are also reported.

Metric	mean ± std	CI 95% (low – high)
Accuracy	0.977878 ± 0.005263	0.975057–0.980434
Balanced Accuracy	0.985024 ± 0.007646	0.981802–0.987812
Macro F1	0.965386 ± 0.008452	0.96081–0.969429
Weighted F1	0.978934 ± 0.005037	0.976282–0.981332
Cohen Kappa	0.97499 ± 0.00594	0.971833–0.977865
MCC	0.975211 ± 0.005871	0.972083–0.978022

#### Experiment 2: Tissue Identification with SmartProbe Single Frequency

**Fig. 6:**
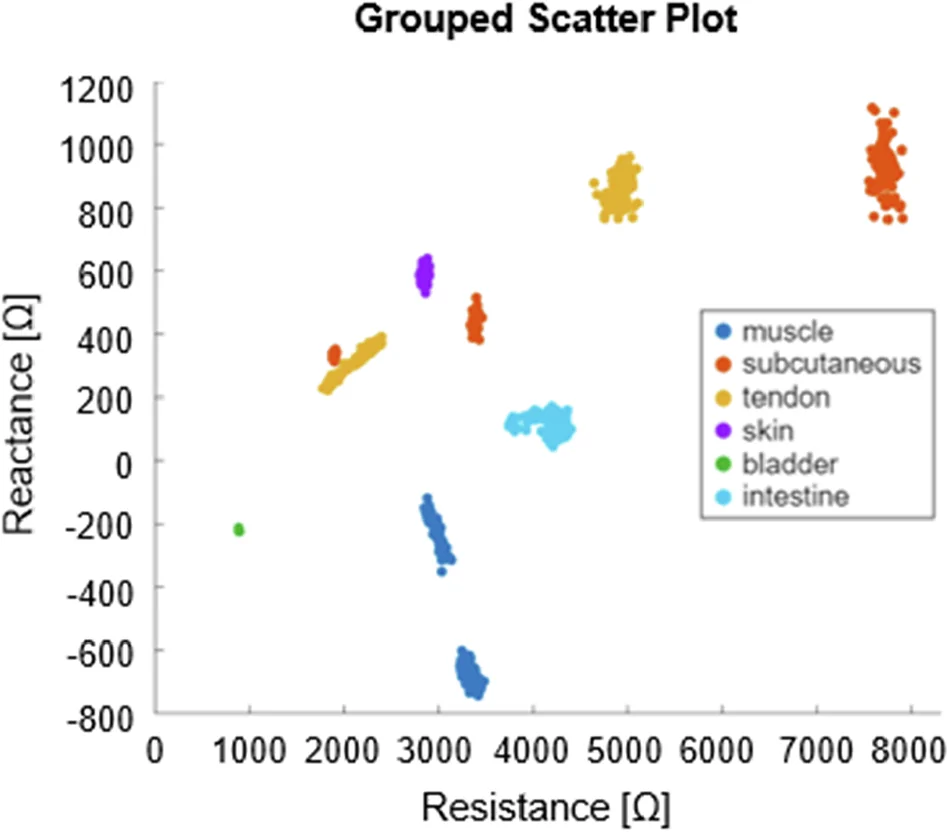
Grouped scatter plot of resistance and reactance of tissues in the subset “B2”.

In this experiment, the goal was to investigate if tissue identification could also be done using EBI data acquired by SmartProbe using its single frequency sensing mode. Specifically, the data used in this experiment was acquired at 100 kHz and corresponds to the subset *Pig1* “B2” used for training and testing. Figure 6 shows the scattering of resistance and reactance values for all the tissues present in this subset.

The clear clustering observed in Fig. 6 for each tissue group indicates that the electrical bioimpedance features provide effective discrimination between biological structures, strongly associated with differences in tissue hydration, cellular organization, vascularization and extracellular matrix composition. Muscle tissue, for example, exhibited comparatively lower resistance values and more negative reactance, reflecting its high water and electrolyte content together with the capacitive contribution of aligned muscle cell membranes. This behavior is consistent with the highly conductive nature of muscular tissue and its anisotropic fiber organization, which facilitates electrical current propagation. Conversely, subcutaneous tissue showed markedly higher resistance values, likely due to its elevated lipid content and lower fluid concentration. Adipose tissue is known to behave as a poor electrical conductor because lipids reduce ionic mobility and limit charge transport through the tissue. Tendon and skin occupied intermediate regions of the feature space. Tendon tissue is characterized by dense collagen-rich fibrous structures and reduced vascularization, factors associated with increased impedance and reduced conductivity. Similarly, the electrical behavior of skin is strongly influenced by the presence of the stratum corneum, which acts as a resistive and capacitive barrier, particularly affecting reactance measurements. Bladder and intestine tissues showed distinct reactance distributions compared with musculoskeletal tissues, likely reflecting differences in tissue architecture, smooth muscle composition and fluid distribution within their walls. In particular, the relatively elevated reactance values observed for bladder tissue may be associated with its layered structure and membrane-related capacitive effects.

Overall, the compactness and separation of the clusters support the hypothesis that bioimpedance measurements capture physiologically meaningful tissue-specific electrical signatures, reinforcing the suitability of the dataset for tissue classification and impedance-based sensing applications.

**Fig. 7:**
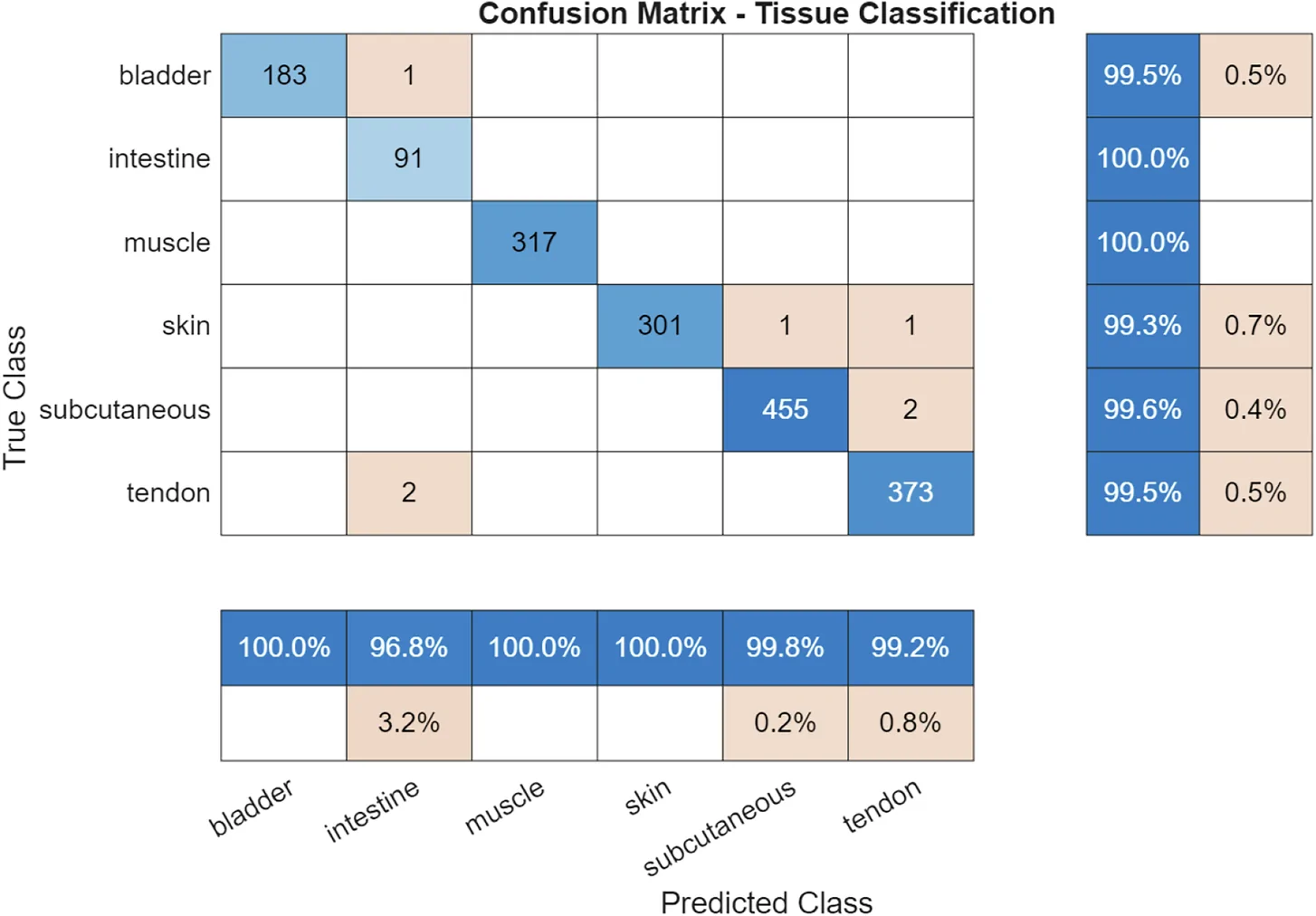
Confusion matrix resulting from tissue identification experiment based on SmartProbe data acquired at 100 kHz.

The Fine KNN model trained and validated on “B2” achieved an overall accuracy of 99.56% and similar values for Recall and F1 score. Table 8 and Table 9 show the performance metrics computed for each class and overall, respectively. Figure 7 illustrates the results on tissue classification for this experiment.

**Table 8:** Class-wise performance metrics of the Fine KNN classifier trained on SmartProbe data acquired at 100 kHz.

Class	Samples	Precision	Recall	Specificity	F1_score
*bladder*	184	1	0.994565	1	0.997275
*intestine*	91	0.968085	1	0.998166	0.983784
*muscle*	317	1	1	1	1
*skin*	303	1	0.993399	1	0.996689
*subcutaneous*	457	0.997807	0.995624	0.999213	0.996714
*tendon*	375	0.992021	0.994667	0.997781	0.993342

**Table 9:** Overall validation performance metrics of the Cubic SVM classifier computed using 5-fold cross-validation predictions on the SmartProbe dataset “B2”. In the right column, 95% confidence intervals are also reported.

Metric	mean ± std	CI 95% (low – high)
Accuracy	0.995947 ± 0.003303	0.992472–0.998842
Balanced Accuracy	0.99638 ± 0.002689	0.993357–0.998967
Macro F1	0.994761 ± 0.005261	0.989627–0.998643
Weighted F1	0.995971 ± 0. 003262	0.992551–0.998843
Cohen Kappa	0.994964 ± 0.0041	0.990673–0.998558
MCC	0.994978 ± 0.004088	0.990689–0.998559

#### Experiment 3: Blood Vessel Puncture Detection with Bipolar electrodes

The goal of this experiment was to investigate if EBI sensing can be used for vessel puncture detection *in vivo*. Data recorded during penetration trials was acquired only with *SmartProbe* using the single frequency sensing modality. This data can be found in the subsets *Pig1* “B1” (acquired at 100 kHz) and *Pig2* “E” (acquired at 20 kHz).

**Fig. 8:**
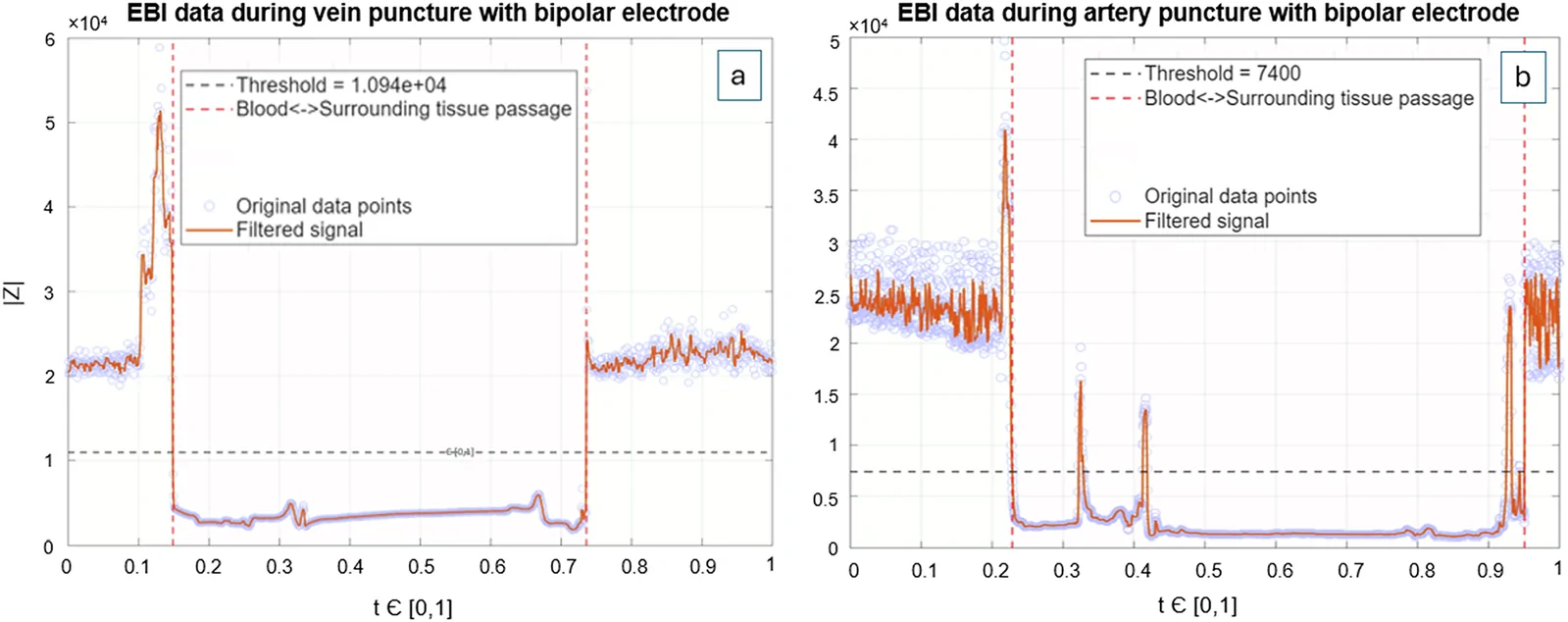
Envelopes of EBI data in subset “B1” (acquired at 100 kHz) highlighting the estimated transition points from surround tissue to blood. (**a**) Venous blood, (**b**) Arterial blood.

**Fig. 9:**
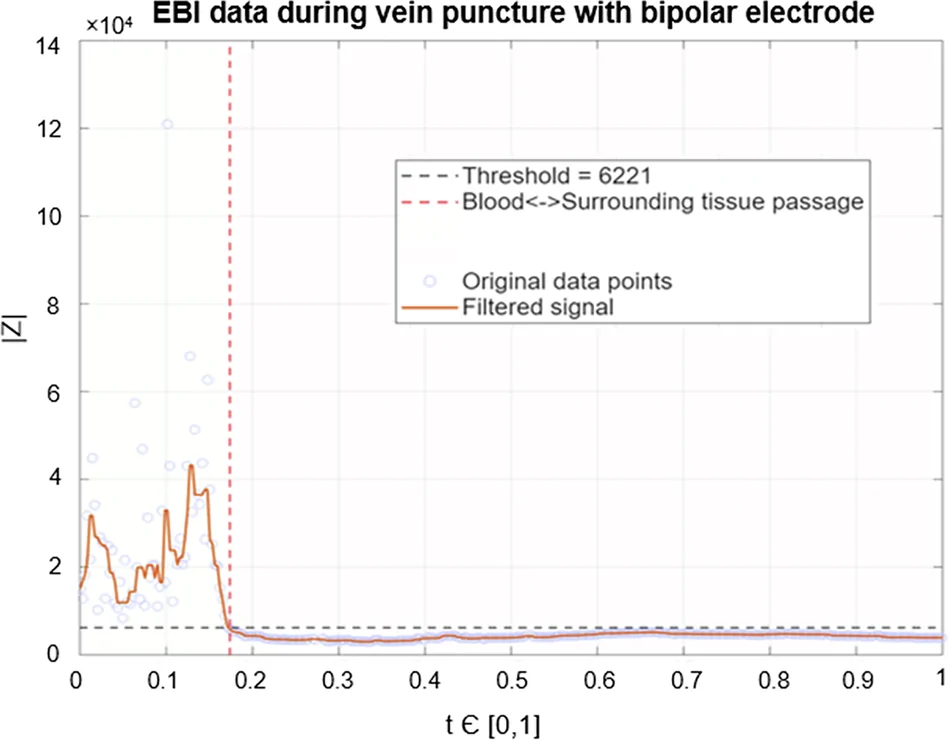
Envelopes of EBI data in subset “E” (acquired at 20 kHz) highlighting the estimated transition points from surround tissue to a venous vessel.

The data processing method implemented in this case consisted in analyzing the envelopes of the EBI signals to identify the points in which the needle passed from surrounding tissue (or air) to blood and vice versa. This was done using an average-based thresholding algorithm capable of discriminating blood from other matters using impedance magnitude values. Examples of such transitions during the penetration of venous and arterial blood vessels are shown in Fig. 8 for subset “B1” and in Fig. 9 for subset “E”.

Subset “B1” was also exploited to evaluate the performance of the threshold-based detection algorithm. To do so, each file was processed individually and compared against the predictions generated by a previously trained Medium KNN classifier. This classifier was selected as it achieved the best performance in the task of distinguishing blood-related signals (arterial and venous blood, from the “B1” subset) from signals originating from the surrounding environment (other tissues and air, from the “B2” subset). For every selected file, data are read and passed to the detect_vesselPuncture function, which applies robust filtering, computes a data-driven threshold and produces a binary segmentation of the signal. In parallel, the same file is processed by the Medium KNN classifier to obtain an independent class prediction for each sample. Finally, a confusion matrix is computed between the threshold-based prediction and the classifier output, from which accuracy is derived. This procedure provides a quantitative assessment of how closely the simple thresholding strategy approximates the behavior of the machine-learning classifier across both single-file and multi-file evaluations. Table 10 shows the results for every data file in “B1”.

**Table 10:** Accuracy of the threshold-based algorithm for the detection of punctures of blood vessels for subset “B1”.

File name	Accuracy of the threshold
pr_202309230950	99.70%
pr_202309231000	97.78%
pr_202309231000	97.86%
pr_202309231030	99.29%
pr_202309231329	91.53%
pr_202309231330	99.37%
pr_202309231334	98.95%
pr_202309231336	99.26%
pr_202309231338	97.66%
pr_202309231339	98.50%
pr_202309231517	94.85%
**Overall**	**97.70%**

#### Experiment 4: Blood Vessel Puncture Detection with Monopolar electrodes

The blood detection task for data acquired with SmartProbe stimulating with a fixed frequency of 100 kHz was performed using data from the *Pig1* subset “C”.

**Fig. 10:**
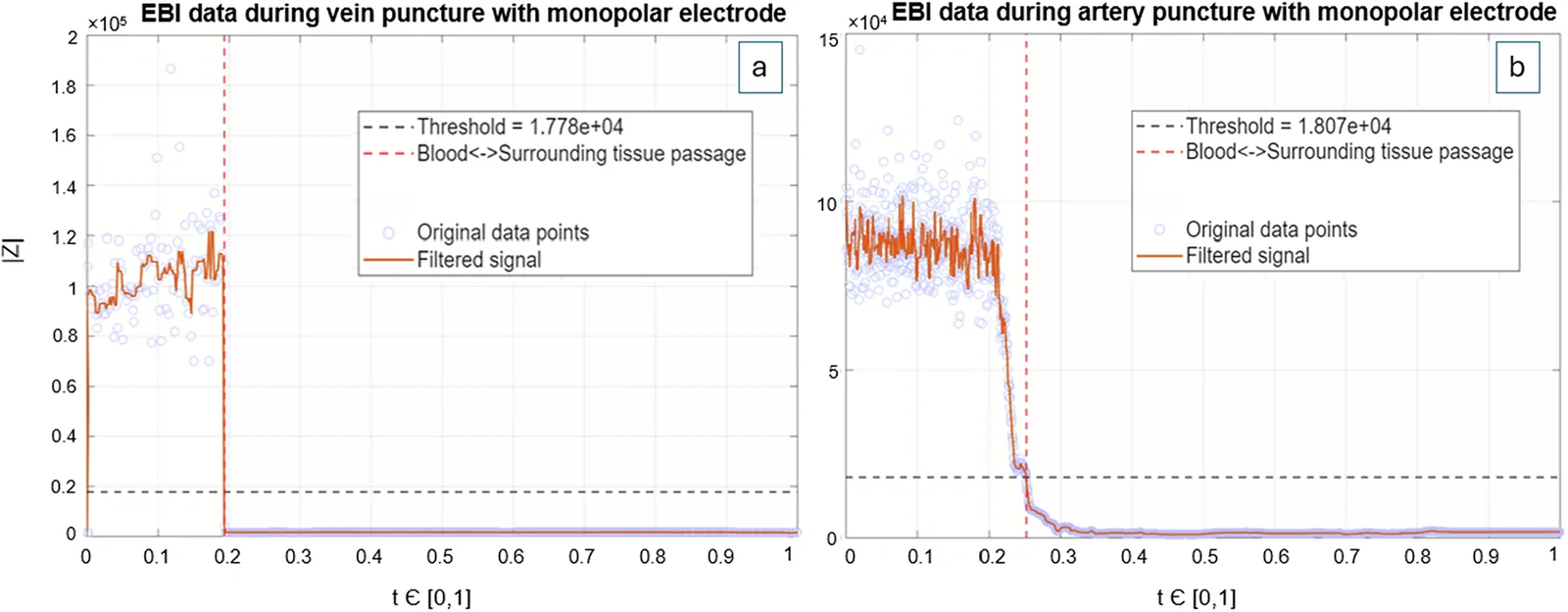
Envelopes of acquired signals in subset “C” highlighting the estimated transition points from surround tissue to blood. (**a**) Venous blood, (**b**) Arterial blood.

Again, the task was evaluated in two phases. The first one consisted in identifying the points of passage from air to blood and vice versa by implementing an average-based thresholding algorithm. An example of air-blood transitions for arterial blood is shown in Fig. 10.

Subset “C” was also exploited to evaluate the performance of the threshold-based detection algorithm. To do so, each file was processed individually and compared against the predictions generated by a Medium KNN classifier previously trained to distinguish values acquired from blood and surrounding tissues. For every selected file, the same procedure as Task 3 is performed. Table 11 shows the results for every data file in “B1”.

**Table 11:** Accuracy of the threshold-based algorithm for the detection of punctures of blood vessels for subset “C”.

File name	Accuracy of the threshold
pr_202309231344	93.93%
pr_202309231345	99.82%
pr_202309231346	100.00%
pr_202309231347	100.00%
pr_202309231348	99.55%
pr_202309231349	100.00%
pr_202309231352	98.66%
pr_202309231354	86.18%
**Overall**	**97.27%**

#### Experiment 5: Recognition of Arterial versus Venous blood with Bipolar electrodes

**Fig. 11:**
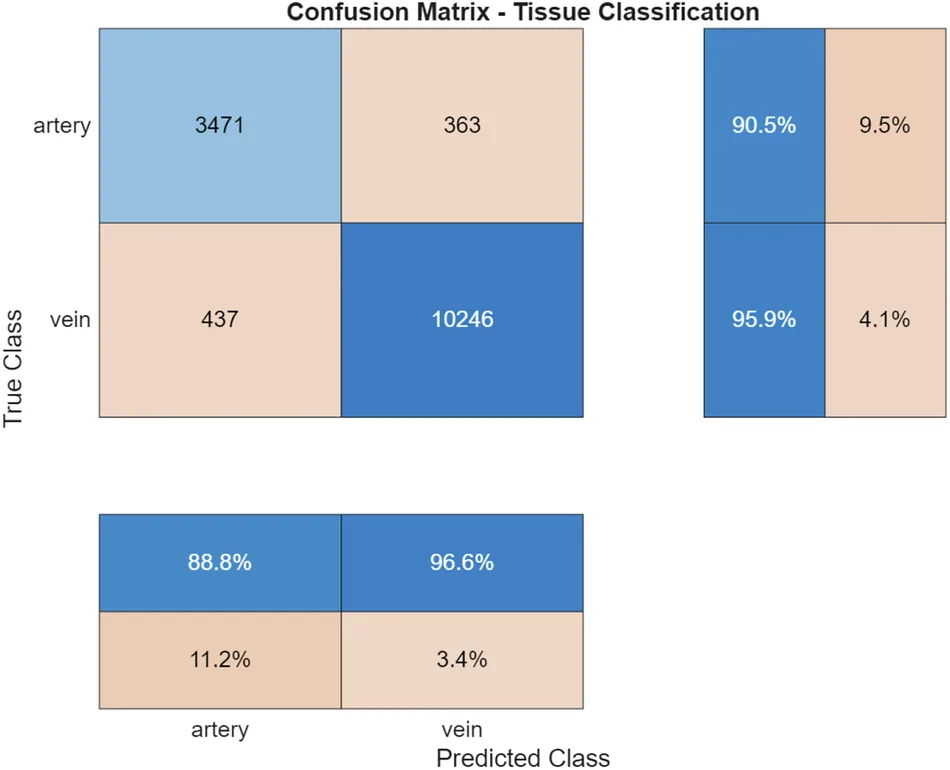
Confusion matrix and metrics resulting from the blood type recognition experiment on subset “B1”.

**Fig. 12:**
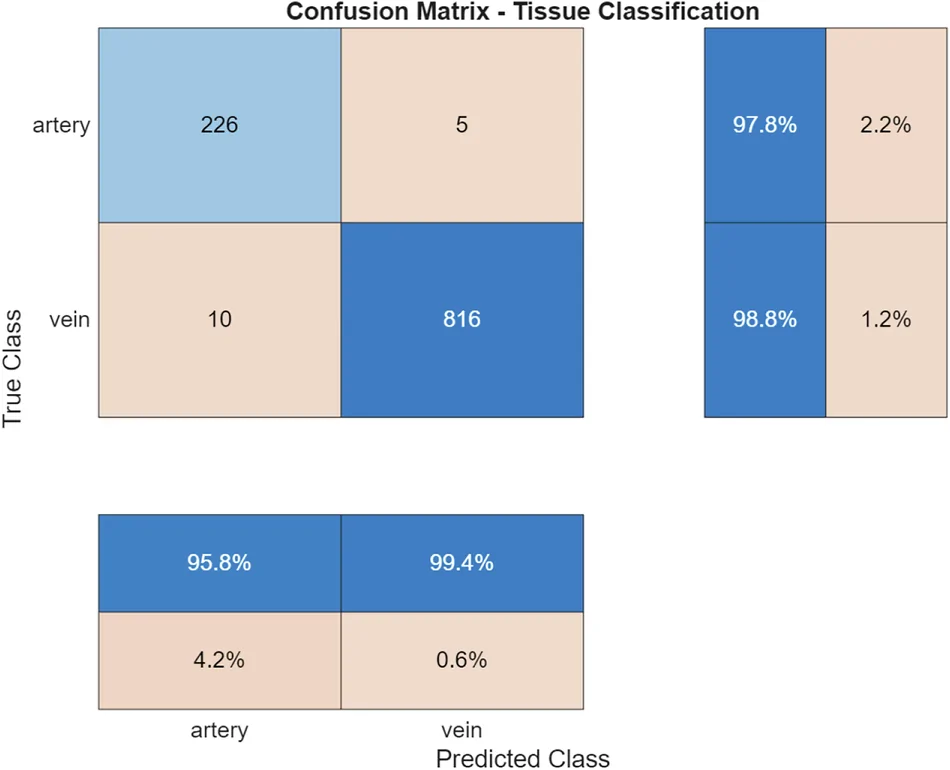
Confusion matrix and metrics resulting from the blood type recognition experiment on subset “E”.

In this experiment, the goal was to investigate the training of a model to discriminate between blood types based on EBI data measured with a bipolar needle electrode. The subsets used were the *Pig1* “B1” (EBI at 100 kHz) and the *Pig2* “E” (EBI at 20 kHz). Both subsets were preprocessed to isolate only the portions of the signal corresponding to blood, removing measurements acquired from surrounding tissues and air. This step was done by applying the dedicated function *wrapper_DataCleaner*; however, it can also be achieved by applying, in sequence, the threshold-based selection algorithm described in the previous experiments. A separate Fine KNN model was trained and validated for each subset given the differences in the signal frequency used for the EBI measurements. The performance metrics for each class and overall are shown in Table 12 and Table 13 for subset “B1”, whereas Table 14 and Table 15 refer to subset “E”. Figure 11 and Fig. 12 illustrate the results on tissue classification for subsets “B1” and “E”, respectively.

**Table 12:** Class-wise performance metrics of the Fine KNN classifier trained on SmartProbe data acquired at 100 kHz.

Class	Samples	Precision	Recall	Specificity	F1_score
*artery*	3834	0.888178	0.905321	0.959094	0.896668
*vein*	10683	0.965784	0.959094	0.905321	0.962427

**Table 13:** Overall validation performance metrics of the Fine KNN classifier computed using 5-fold cross-validation predictions on the SmartProbe dataset “B1”. In the right column, 95% confidence intervals are also reported.

Metric	mean ± std	CI 95% (low – high)
Accuracy	0.944892 ± 0.003783	0.941241–0.948715
Balanced Accuracy	0.932206 ± 0.005095	0.927153–0.937456
Macro F1	0.929548 ± 0.004766	0.925012–0.934458
Weighted F1	0.94506 ± 0.003736	0.941438–0.948923
Cohen Kappa	0.859104 ± 0.009529	0.85003–0.868919
MCC	0.859226 ± 0.009509	0.850121–0.868976

**Table 14:** Class-wise performance metrics of the Fine KNN classifier trained on SmartProbe data acquired at 20 kHz.

Class	Samples	Precision	Recall	Specificity	F1_score
*artery*	231	0.957627	0.978355	0.987893	0.96788
*vein*	826	0.99391	0.987893	0.978355	0.990893

**Table 15:** Overall validation performance metrics of the Fine KNN classifier computed using 5-fold cross-validation predictions on the SmartProbe dataset “E”. In the right column, 95% confidence intervals are also reported.

Metric	mean ± std	CI 95% (low – high)
Accuracy	0.985804 ± 0.004751	0.97824–0.992431
Balanced Accuracy	0.983127 ± 0.007236	0.973028–0.991808
Macro F1	0.979382 ± 0.006905	0.969003–0.989187
Weighted F1	0.985865 ± 0.004716	0.978427–0.992442
Cohen Kappa	0.958767 ± 0.013809	0.938011–0.978374
MCC	0.958997 ± 0.013734	0.938233–0.978407

#### Experiment 6: Recognition of Arterial versus Venous blood with Monopolar electrodes

**Fig. 13:**
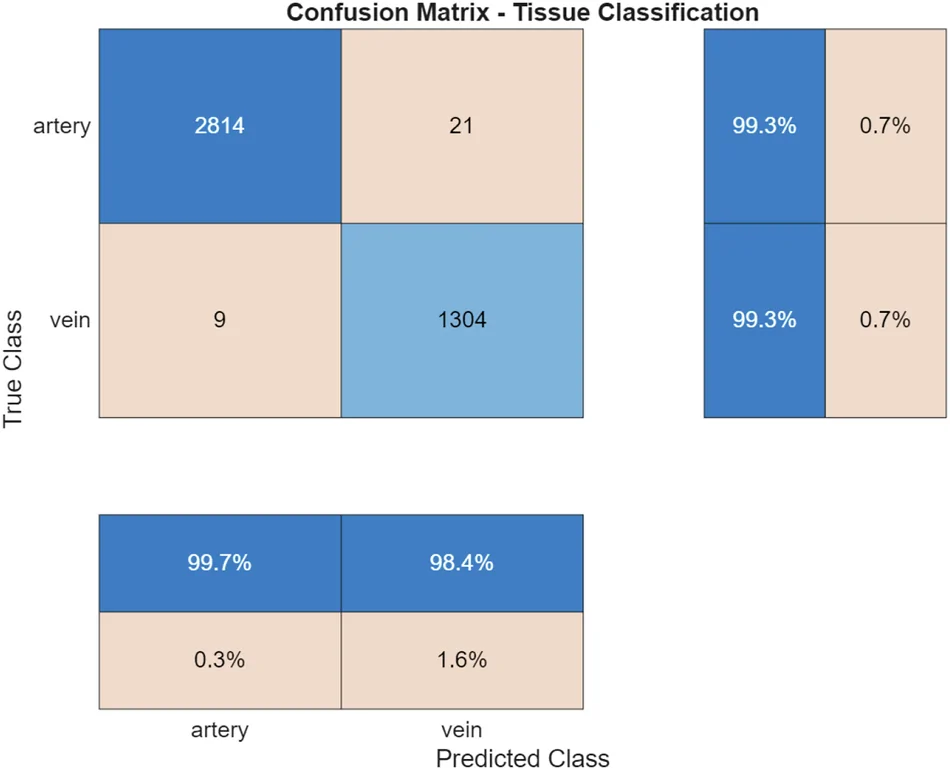
Confusion matrix and metrics resulting from the blood type recognition experiment on subset “C”.

As in the previous experiment, the goal here was to investigate the automatic recognition of different blood types based EBI sensing, which in this case was performed using a monopolar needle electrode. For this, the subset used was the *Pig1* “C”, which contains EBI measurements acquired with *SmartProbe* at a fixed stimulating frequency of 100 kHz. The subset was preprocessed to isolate only the portions of the signal corresponding to blood, removing measurements acquired from surrounding tissues and air with a dedicated function. The Fine KNN model trained and validated on this data achieved a validation Accuracy of 99.5%. Table 16 and Table 17 show the performance metrics computed for each class and overall, respectively. Figure 13 illustrates the results on tissue classification for this experiment.

**Table 16:** Class-wise performance metrics of the Fine KNN classifier trained on SmartProbe data in subset “C”.

Class	Samples	Precision	Recall	Specificity	F1_score
*artery*	2835	0.996812	0.992593	0.993145	0.994698
*vein*	1313	0.984151	0.993145	0.992593	0.988628

**Table 17:** Overall validation performance metrics of the Fine KNN classifier computed using 5-fold cross-validation predictions on the SmartProbe dataset “C”. In the right column, 95% confidence intervals are also reported.

Metric	mean ± std	CI 95% (low – high)
Accuracy	0.992767 ± 0.002831	0.989875–0.995178
Balanced Accuracy	0.992873 ± 0.002367	0.989763–0.995294
Macro F1	0.991668 ± 0.003233	0.988458–0.994376
Weighted F1	0.992779 ± 0.002812	0.9899–0.995181
Cohen Kappa	0.983336 ± 0.006464	0.976918–0.988751
MCC	0.983398 ± 0.006377	0.976947–0.988761

### Dataset limitations

Some limitations of the present dataset should be acknowledged. First, the signals were acquired from only two porcine subjects under controlled experimental conditions. Therefore, the generalizability of the reported measurements across different animals, physiological conditions or species cannot be fully established. Biological variability in tissue electrical properties may arise from several factors, including age, hydration state, vascularization, anatomical variability and surgical conditions, which are not exhaustively represented in the current dataset.

In addition, the limited number of samples available for some tissue categories may affect the representativeness of the dataset and reduce the ability to fully capture the natural variability of *in vivo* bioimpedance measurements. Consequently, although the dataset provides a structured and diverse collection of EBI signals suitable for methodological development and comparative analyses, caution should be exercised when extrapolating the observed electrical characteristics to broader populations or different experimental settings.

The limited sample size might also impact on the interpretation of the classification results presented in this work. While the reported performance metrics demonstrate clear separability among several tissue classes within the acquired data, the classifiers were trained and validated on a relatively restricted number of acquisitions and biological subjects. Therefore, the obtained accuracies should primarily be interpreted as a technical validation of the dataset consistency and of the feasibility of EBI-based tissue discrimination, rather than as definitive evidence of clinical-level generalization.

The proposed dataset may be expanded in future studies by including a larger number of animal subjects, additional tissue categories and pathological conditions. Moreover, extending the frequency range and incorporating data acquired with different sensing devices and electrode configurations would further improve the representativeness of the dataset and support more robust evaluation and benchmarking of EBI-based approaches.

## Data Availability

The *in vivo* tissue bioimpedance dataset was deposited into Zenodo and can be accessed at the following link: https://doi.org/10.5281/zenodo.18769576. It is organized in a hierarchical folder structure based on subject identity, measurement configuration and acquisition type. Data were collected from two animals and stored as .txt files including a metadata header describing the acquisition device and settings, followed by columns with measurement mode and raw EBI values.

## Code availability

Custom MATLAB scripts used to process, analyze, and validate the dataset are provided in the folder “*Tissue Analysis Scripts*”. These include functions for data import, preprocessing, SMOTE-based class balancing and classification. All code was developed and tested using MATLAB R2025b. No proprietary toolboxes were used beyond those available in standard MATLAB installation. A description of the full content of the folder is provided in the “Supplementary” section.
